# Supplementary material for: HIVprotI: an integrated web based platform for prediction and design of HIV proteins inhibitors
Source: J Cheminform. 2018 Mar 9;10:12. doi: 10.1186/s13321-018-0266-y (PMC5845081; doi:10.1186/s13321-018-0266-y)
Supplement: Supplementary file 2 — Additional file 2. Source code of HIVProtI web server. [file 13321_2018_266_MOESM2_ESM.zip › HIVprotI_Source-code/hivprotI/weka-3-6-6/doc/weka/classifiers/functions/neural/NeuralMethod.html]

NeuralMethod


---


|  |  |  |
| --- | --- | --- |
| **Overview** \| **Package** \| **Class** \| **Tree** \| **Deprecated** \| **Index** \| **Help** \| **Weka's home** | |  |
| **PREV CLASS**   **NEXT CLASS** | **FRAMES**    **NO FRAMES**     **All Classes** |  |
| SUMMARY: NESTED \| FIELD \| CONSTR \| METHOD | DETAIL: FIELD \| CONSTR \| METHOD |  |


---


## weka.classifiers.functions.neural Interface NeuralMethod

**All Superinterfaces:**: java.io.Serializable

**All Known Implementing Classes:**: LinearUnit, SigmoidUnit

---

``` public interface NeuralMethod extends java.io.Serializable ```

This is an interface used to create classes that can be used by the
neuralnode to perform all it's computations.

**Version:**
:   $Revision: 1.6 $

**Author:**
:   Malcolm Ware (mfw4@cs.waikato.ac.nz)

---

| **Method Summary** | |
| --- | --- |
| `double` | `errorValue(NeuralNode node)`             This function calculates what the error value should be. |
| `double` | `outputValue(NeuralNode node)`             This function calculates what the output value should be. |
| `void` | `updateWeights(NeuralNode node, double learn, double momentum)`             This function will calculate what the change in weights should be and also update them. |

**Method Detail**

### outputValue

```
double outputValue(NeuralNode node)
```

:   This function calculates what the output value should be.

    :   **Parameters:**: `node` - The node to calculate the value for. **Returns:**: The value.

---


### errorValue

```
double errorValue(NeuralNode node)
```

:   This function calculates what the error value should be.

    :   **Parameters:**: `node` - The node to calculate the error for. **Returns:**: The error.

---


### updateWeights

```
void updateWeights(NeuralNode node,
                   double learn,
                   double momentum)
```

:   This function will calculate what the change in weights should be
    and also update them.

    :   **Parameters:**: `node` - The node to update the weights for.: `learn` - The learning rate to use.: `momentum` - The momentum to use.


---


|  |  |  |
| --- | --- | --- |
| **Overview** \| **Package** \| **Class** \| **Tree** \| **Deprecated** \| **Index** \| **Help** \| **Weka's home** | |  |
| **PREV CLASS**   **NEXT CLASS** | **FRAMES**    **NO FRAMES**     **All Classes** |  |
| SUMMARY: NESTED \| FIELD \| CONSTR \| METHOD | DETAIL: FIELD \| CONSTR \| METHOD |  |


---
